# Neurocognitive Empowerment for Addiction Treatment (NEAT): study protocol for a randomized controlled trial

**DOI:** 10.1186/s13063-021-05268-8

**Published:** 2021-05-07

**Authors:** Hamed Ekhtiari, Tara Rezapour, Brionne Sawyer, Hung-Wen Yeh, Rayus Kuplicki, Mimi Tarrasch, Martin P Paulus, Robin Aupperle

**Affiliations:** 1 Laureate Institute for Brain Research, 6655 South Yale Ave., Tulsa, OK 74136 USA; 2 Institute for Cognitive Science Studies, Tehran, Iran; 3 Children’s Mercy Hospital, Kansas City, MO USA; 4 Family and Children Services, Tulsa, OK USA

**Keywords:** Opioid, Methamphetamine, Neurocognitive deficits, Meta-cognition, Rehabilitation, Awareness, Addiction, Substance use disorder, Diversion program, Women

## Abstract

**Background:**

Neurocognitive deficits (NCDs) and associated meta-cognition difficulties associated with chronic substance use often delay the learning and change process necessary for addiction recovery and relapse prevention. However, very few cognitive remediation programs have been developed to target NCDs and meta-cognition for substance users. The study described herein aims to investigate the efficacy of a multi-component neurocognitive rehabilitation and awareness program termed “Neurocognitive Empowerment for Addiction Treatment” (NEAT). NEAT is a fully manualized, cartoon-based intervention involving psychoeducation, cognitive practice, and compensatory strategies relevant across 10 major cognitive domains, including aspects of attention, memory, executive functions, and decision-making.

**Method/design:**

In a single-blind randomized controlled trial (RCT), 80 female opioid and/or methamphetamine users will be recruited from an addiction recovery program providing an alternative to incarceration for women with substance use-related offenses. Eight groups of 9–12 participants will be randomized into NEAT or treatment-as-usual (TAU). NEAT involves 14 90-min sessions, delivered twice weekly. The primary outcome is change in self-reported drug craving from before to after intervention using Obsessive Compulsive Drug Use Scale. Secondary and exploratory outcomes include additional psychological, neurocognitive, and structural and functional neuroimaging measures. Clinical measures will be performed at five time points (pre- and post-intervention, 3-, 6-, and 12-month follow-up); neuroimaging measures will be completed at pre- and post-intervention.

**Discussion:**

The present RCT is the first study to examine the efficacy of an adjunctive neurocognitive rehabilitation and awareness program for addiction. Results from this study will provide initial information concerning potential clinical efficacy of the treatment, as well as delineate neural mechanisms potentially targeted by this novel intervention.

**Trial registration:**

ClinicalTrials.govNCT03922646. Registered on 22 April 2019

## Background

Opioid use disorder (OUD) and methamphetamine use disorder (MUD) are among the costliest mental health disorders in the USA and worldwide [1, 2]. Opioids (including prescription pain relievers and the illicit drug heroin) were involved in more than 42,000 deaths in the USA in 2016, more than any year on record [3]. This accounts for more deaths than road accidents and gun violence combined. In 2017, amphetamine use disorders were reported as the second most common use disorders worldwide by the United Nations Office on Drugs and Crimes (UNODC) [4]. Even more alarming, the number of overdoses with heroin and methamphetamine has tripled from 2011 to 2016 [5].

Chronic OUD and MUD are associated with numerous other mental health symptoms, including anxiety and depression [6, 7], as well as subjective and objective neural and cognitive deficits. These neurocognitive deficits (NCDs) are apparent across domains of memory, attention, decision-making, judgment, negative affect, metacognition, self-regulation, and impulse inhibition [8–10]. Neuroimaging studies indicate that these deficits may relate to dysfunction within a broad network of the brain regions, including the prefrontal cortex, orbitofrontal cortex, amygdala, nucleus accumbens, hippocampus, and anterior cingulate gyrus [11–13]. Maladaptive neural changes in these regions result in aberrant salience attribution, increased reward expectancy, changed learning pattern (including conditioned-incentive learning, habit learning, declarative learning), and impulsive decision-making [13].

Current treatment programs for OUDs and MUDs are mainly focused on abstinence from illicit drugs, with the assumption that the NCDs will be subsequently restored. However, NCDs are found to persist or sometimes even become exacerbated after long-term abstinence and are thought to contribute to relapse, decreased quality of life, and/or lack of reintegration into society [14]. Furthermore, NCDs (particularly those related to attention and memory) are considered a potential predictor for treatment outcomes for either the core substance use disorder or co-occurring symptoms (i.e., cognitive-behavioral therapies) [15, 16].

Numerous cognitive rehabilitation programs have been developed to focus on compensatory strategies and restorative exercises for NCDs associated with traumatic brain injuries [17], stroke [18], multiple sclerosis [19], and schizophrenia [20]. These programs have consistently been found to improve functioning and long-term outcomes for these groups of patients. There have been a few preliminary attempts to transplant cognitive rehabilitation programs for use with substance use populations, providing initial evidence that such programs could be beneficial. However, these programs did not explicitly link the cognitive rehabilitation modules to the processes involved in addiction and recovery. There is potential value in clarifying how such strategies may relate to the current neuroscientific understanding of addiction and recovery, as well as the co-occurring symptoms often experienced.

The present pilot study aims to characterize the clinical potential for an intervention targeting NCDs in OUDs and MUDs by enhancing awareness and use of neurocognitive skills in the context of substance use recovery. This aim will be accomplished by randomizing subjects who are already enrolled in substance use treatment to complete a novel “Neurocognitive Empowerment for Addiction Treatment” (NEAT) program or treatment-as-usual intervention. NEAT is novel in (a) its use of cartoons, brain awareness games, and real-life scenarios to ensure it is interactive and engaging; (b) its focus on the role of neurocognitive deficits in recovery from substance use and co-occurring mental health symptomatology; and (c) its incorporation of neuroscience findings specific to substance use.

The primary outcome in this trial is “change in self-reported drug craving from baseline to after intervention using the Obsessive Compulsive Drug Use Scale (total score) [21]. Secondary outcome is the mean change in self-report subjective cognitive functioning from baseline to after intervention using PROMIS Cog Abilities [22] and PROMIS Cog General measures [22]. Exploratory outcomes include additional psychological, neurocognitive, and structural and functional neuroimaging measures and are available on ClinicalTrials.gov. We hypothesis that as compared to the active control, the NEAT program will be associated with the following:

1. Greater reduction in self-report drug carving symptom from before to after intervention (primary outcome)
2. Enhanced subjective cognitive functioning from before to after intervention (secondary outcome)
3. Improved objective neuropsychological functioning from before to after intervention
4. Reduced mental health (i.e., depression and anxiety) symptoms from before to after intervention and other follow-up time points
5. Lower number of relapses as measured with urine drug tests during follow-up time points
6. Decreases in subcortical limbic (amygdala and ventral striatum) activation and increase prefrontal cortical top-down inhibitory activation during induced drug craving in fMRI drug cue reactivity from before to after intervention
7. Increases in cortical gray matter density (measured with voxel-wise brain morphometry) and white matter track integrity (measured with fractional anisotropy) in areas related to self-control and awareness, including prefrontal gray matter and frontostriatal/frontoamygdalar white matter from before to after intervention

Outcome measures 3 to 7 are exploratory.

## Methods/design

### Study objectives

The present study aims to (a) determine the potential clinical impact of the NEAT program on clinical symptoms and function, from pre- to post-intervention and at long-term follow-up (12 months after treatment completion), and (b) explore the potential impact of the NEAT program on functional and structural brain recovery, from pre- to post-intervention.

### Research environment/context

The present trial is conducted by Laureate Institute for Brain Research (LIBR), and participants are recruited from a single site, the Women in Recovery (WIR) program at Family and Children’s Services located in Tulsa, OK. WIR is an intensive outpatient alternative for eligible women facing long prison sentences for non-violent drug-related offenses.

### Design

**Fig. 1:**
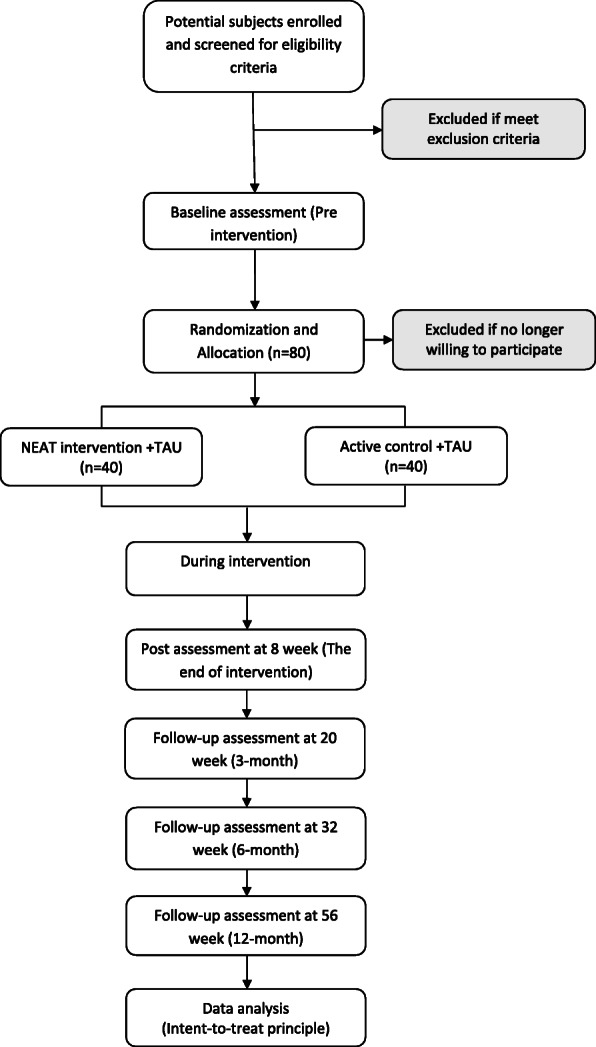
CONSORT flow diagram. The flow chart depicts participant progression through the study from the initial enrolment to allocation, follow-up, and finally analyses of their data stages. Planned sample size, *n* = 64; to be enrolled, *n* = 80. Forty participants will be enrolled in each arm (expected to be 8 dropouts in each arm from pre- to post-intervention on average)

This is an assessor-blinded, two-arm, parallel-group, randomized controlled clinical trial formulated in accordance with the Consolidated Standards of Reporting Trials (CONSORT) 2010 guidelines (supplementary file 1). This protocol was written using the Standard Protocol Items: Recommendations for Interventional Trials (SPIRIT) guidelines, and the SPIRIT checklist is provided in supplementary file 2. Eighty participants will be recruited in eight groups. Groups will be randomly allocated to the intervention (*n* = 40) and control (*n* = 40) groups in blocks of 4 in a 1:1 ratio. All 80 participants will receive treatment-as-usual (TAU). Those subjects allocated to the intervention group will receive TAU but will complete NEAT in lieu of nonessential aspects of the TAU program that do not explicitly target neurocognitive function (e.g., culinary or legal education classes). Thus, both NEAT and TAU groups received similar amount (e.g., intensity, duration, frequency) of intervention. Self-report, interview-based, and neuroimaging assessments will be performed before (pre; week 0), during intervention (from week 1 to week 7), after the intervention period (post; week 8), and/or after a follow-up period (3, 6, 12 months; weeks 20, 32, 56, respectively). Interview-based assessments were conducted by a trained clinical practitioner blind to the allocation of participants. Figure 1 summarizes the full design of the trial in accordance with the CONSORT flow diagram.

The trial was approved by the Western Institutional Review Board (approval number: 20181028, 1-1141403-1) and registered in ClinicalTrials.gov on 22 April 2019 with NCT03922646 as ClinicalTrials.gov identifier no.; amendments have been made to the protocol since the original submission to ClinicalTrials.gov. The study is funded by the Oklahoma Center for Advancement of Science and Technologies (OCAST) Hamed Ekhtiari [HE], principal investigator [PI], and Robin Aupperle [RA] as co-investigator [CI].

As this study is considered a “no or minimum risk” trial, there is no independent data monitoring committee (DMC) assigned for this trial. Trial investigators will monitor the data collection process, fidelity of the intervention, and integrity of protocol implementation on regular weekly basis meetings with the research staff. There is no plan for interim analyses, and there are no anticipated formal stopping rules for the trial as there are no anticipated problems that could be detrimental to the participants or the study integrity. The data that will be collected in this study will be available from the principal investigator on reasonable request.

### Participants

#### Inclusion criteria

To participate in this trial, subjects must (1) be 18 and 55 years old; (2) enrolled in WIR within 6 months; (3) diagnosed with OUD and/or MUD (based on the Mini-International Neuropsychiatric Interview (MINI) diagnostic interview for DSM-5 [23]) over the last 12 months; (4) be able to provide written informed consent; (5) have sufficient proficiency in the English language to understand and complete interviews, questionnaires, and all other study procedures; and (6) have completed at least an 8th grade education, to help facilitate the ability to engage in the written materials included in the NEAT program.

#### Exclusion criteria

Potential subjects will be excluded if they (1) are reluctant or unable to complete any of the major aspects of the study protocol, including self-report or behavioral assessment; (2) have uncorrectable vision or hearing problems that would interfere with the completion of the study procedures; (3) have diagnoses of schizophrenia spectrum or other psychotic disorders; (4) have current mental or physical health symptoms that require immediate attention (such as suicidal ideation with intent or plan, active psychotic symptoms, delirium); (5) have a history of unstable liver or renal insufficiency; glaucoma; significant and unstable cardiac, vascular, pulmonary, gastrointestinal, endocrine, neurologic, hematologic, rheumatologic, or metabolic disturbance; or any other condition that, in the opinion of the investigator, would make participation not be in the best interest (e.g., compromise the well-being) of the subject or that could prevent, limit, or confound the protocol-specified assessments; (6) have a positive test for drugs of abuse at the time of baseline assessment, including alcohol, cocaine, marijuana, opiates, amphetamines, methamphetamines, phencyclidine, benzodiazepines, barbiturates, methadone, and oxycodone; (7) have MRI contraindications; and (8) have moderate to severe traumatic brain injury (loss of consciousness for less than 30 min or > 24 h posttraumatic amnesia) or other neurocognitive disorder with evidence of neurological deficits, neurological disorders, or severe or unstable medical conditions that might be compromised by participation in the study. There is no limitation in the medications that participants could receive before, during, and after intervention. However, the list of medication will be recorded for exploratory analysis.

### Sample size calculation

With total enrollment of *N* = 80 and an estimated 20% attrition rate, we anticipate having complete data at our primary endpoint (post-treatment) for 32 subjects per group or 64 in total. Due to cluster randomization, the effective sample size will be *n* = (*mk*)/[1 + *ρ*(*m* − 1)] where *m*, *k*, *ρ* are the number of subjects per cluster, the number of clusters, and the intra-cluster correlation, respectively [24]; here, *k* = 4, and on average, *m*= 8 (80% of 10) per cluster. Considering a range of 0.05 to 0.3 for *ρ*, *n*_Eff_ will be somewhere between 10.3 and 23.7 per group. This effective sample size will provide a precision of *t*_*n*-1,1-*α/2*_/√*n* or 0.43–0.70, times standard deviation as margin of error at 95% confidence for a continuous measure (self-reported drug craving), and 80% power to detect large effect sizes (*d* = 0.83 to 1.3) for group differences in pre to post or pre to follow-up changes at a two-sided 5% significance of a 2-sample *t* test. While we may be underpowered to detect small to medium effects, the current pilot study will be crucial for informing the viability of the intervention and for estimating power for future, larger clinical trials.

### Recruitment

Study participants will be recruited through the WIR program at Family and Children’s Services. Flyers will be provided to the clinic program staff to be given to potential subjects. The research team may also discuss the study to the groups of patients (i.e., when gathered for a class). The research team will be available at WIR on a regular basis to discuss the study privately with subjects interested in the study. The WIR staff will not be present during these individual meetings in order to increase confidentiality and ensure that subjects are making an informed decision to participate. All efforts will be made to ensure that the recruited subject population closely resembles the ethnic and racial composition of the WIR program. The enrolled participants then will give a written informed consent to one of the principle investigators (PI) or research coordinator (RC) in a private interview/exam room at LIBR or WIR. Family members will be allowed to be present and discuss the consenting process with the participant if requested. In this session, it will be emphasized that participation is strictly voluntary and that they have the right to withdraw at any time without penalty. It will be emphasized that the information provided during the course of the study will have no impact on participants’ treatment and care provided by WIR or other programs and clinics, their experience at correctional facilities, or on any court processes or decisions. Follow-up meetings with the WIR staff and directors will be held to make sure the reasonable number of recruitments will happen to meet the planned sample size.

Note that participants will be monetarily compensated for their time of participation in self-report, behavioral, and neuroimaging assessment. Each participant will be also provided with a gift card ($10–15 per session) to compensate completing intervention session assessments.

There are three follow-up assessment sessions 3, 6, and 12 months after completion of the intervention with urine drug test and questionnaires. Participants will be compensated for their time for the follow-up assessments as well.

### Randomization and blinding

All consented participants will receive NEAT or other active, but non-essential regularly scheduled programming at WIR coinciding with the same timeframe. Randomization will be happening in the group level, and groups of 9–12 participants will be randomly allocated to intervention conditions (study arms). This randomization will be conducted in blocks of 4 groups. The randomization will be determined prior to enrolling any subjects using computer-generated allocations.

The randomization codes will be generated by a statistician not involved in enrollment or delivery of interventions and will be kept in a secure computer file with controlled access. The research staff involved in the conduct of interview-based assessments (e.g., MINI, PRISM-5) will be blinded to the intervention condition. Participants and all research staff are kept blind to their intervention condition until completion of all baseline assessments. However, due to the nature of the intervention, participants and research staff involved in providing interventions will not be kept blind to the interventions after completion of baseline assessments. Research investigators (who are unblinded) will be involved in both delivering the intervention and doing the data analysis; therefore, outcome analysis will not be blinded.

### Treatment-as-usual

All participants will complete the essential components of the WIR treatment program. The WIR program includes daily and weekly clinical visits and both individual and group sessions. These sessions include but are not limited to cognitive behavioral therapy, cognitive processing therapy, motivational interviewing, behavioral modification and strength-based case management interventions, nutritional education, and occupational training. The WIR program is conducted in three phases, with increasing independence at each phase (e.g., supervised probation with ankle monitor and residing in program-supervised housing at first; independent housing, independent employment, and outpatient visits multiple times per week towards the end of the program). Phase 1 includes 40 structured hours weekly that is devoted to addiction and trauma treatment, parent education, job readiness training, life skills, safe group housing, education, and community volunteering. Phase 2 includes 24 structured hours weekly that is devoted to job retention training, part-time job replacement, parent-child visitation, health and wellness services, education groups, subsidized housing, financial literacy, and recovery support. Phase 3 includes 12 structured hours weekly that considered full-time employment, parent-child reunification, education groups, independent housing, recovery support technology, and community integration. Graduation from the program occurs after completion of phase 3, an average of 17 months in the program. Random drug testing is conducted by WIR throughout participation in the program. After graduation, women can continue participating in group programming on a volunteer basis. Implementing active or active-control interventions will not require alteration to treatment-as-usual.

### Interventions

#### Active control intervention

Participants in the comparison group will receive the essential components of the WIR program (TAU) plus additional WIR training sessions related to health and wellness, communication skills, and/or financial literacy, scheduled at the same days/times as the NEAT sessions. Thus, the active control intervention has the same duration and frequency as the active intervention without involving the cognitive training content designed for the active intervention.

#### Active intervention

**Fig. 2:**
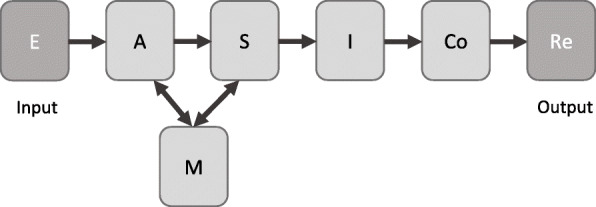
Dynamic model of craving (DCM). A neuroscience-informed conceptual framework to define the major cognitive targets in the dynamic response to drug-related or emotionally salient cues that can end up to drug-seeking and consumption behaviors. E, environment; A, attention, S, saliency processing; M, memory; I, interoception; Co, control; R, response

The active intervention in this trial, the Neurocognitive Empowerment for Addiction Treatment (NEAT), was developed to target cognitive domain thoughts to be either influenced by substance use or involved in drug craving and seeking behavior based on the dynamic model of craving (DCM) (Rezapour et al. [25]). According to the DCM model (Fig. 2) which has been inspired by the traditional cognitive behavioral therapy frameworks [26], drug craving is triggered by environmental cues (E) (including both internal and external cues). These cues then elicit distinct yet collaborative bottom-up and top-down attentional processes that increase the focus of attention on drug-relevant cues (A). This attentional deployment activates saliency evaluation processes (S) related to drug-associated cues. The retrieval of memories (M) linked to the drug cues provides relevant information during saliency processing. Saliency evaluation can result in an interoceptive state that represents the subjective feeling of craving, leading to the engagement of executive control (Co) and decision-making processes to execute actions supporting drug-taking behavior or abstinence. In targeting these functions, NEAT also provides skills important for daily functioning and re-integration into the society, including self-monitoring, time management, and problem solving [27].

NEAT consists of 14 90-min group sessions, which will be led by two trained master’s level therapists or therapists-in-training (e.g., doctoral students in clinical psychology) supervised by a licensed clinical psychologist (RLA). Groups are led by therapists according to the session-by-session manual. All sessions will be audio-recorded to support supervision and fidelity of implementation. Cartoon-based treatment binders and brain planners are provided to all participants. As the intervention is designed to be delivered in the group therapy setting during the trial and also in future clinical use, clustering of the effect due to the group administration of the intervention is not accounted as a variable of interest.

**Fig. 3:**
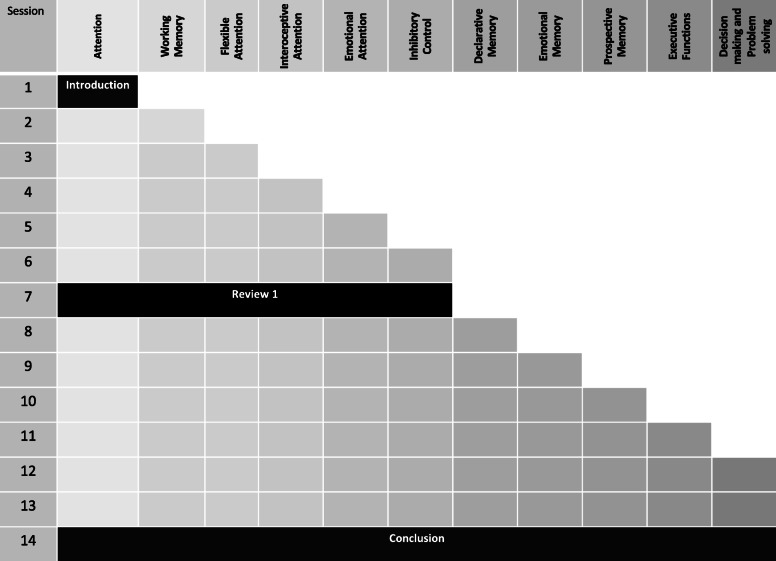
Architecture of Neurocognitive Empowerment for Addiction Treatment (NEAT) for 14 sessions. In each session, a new cognitive domain is added to the previous one in trainings and exercises (accumulative architecture). The brightness of color indicates the level of complexity of the cognitive modules. The basic modules (e.g., sustained attention) marked with bright colors, and the complex ones marked with dark colors (e.g., monitoring)

Each session starts with a didactic, psychoeducation portion describing the concepts and skills of focus in each session. The session is followed by a written material with descriptions of the concepts/skills in verbal and pictorial (cartoon) formats. The session continues with practicing cognitive tasks/games relevant for each skill and ends with a discussion of how they can monitor and practice each skill in their everyday life. Besides the in-session components, subjects are provided with homework material to practice the learned objectives between sessions, including monitoring their use of various brain functions, implementing compensatory strategies, and completing additional cognitive exercises. They are also asked to complete aspects of the brain planner between each session, starting with the retrospective recording of events and slowly progressing towards prospective planning. NEAT will target the cognitive domains shown in Fig. 3 through the use of the following five components:

1. Neuroscience-informed psychoeducation: The main purpose of the neuroscience-informed psychoeducation component is to promote subjects’ awareness about the cognitive impairments often associated with drug craving and addiction. Participants are provided with information concerning the common behavioral manifestations of these impairments in daily life activities. They are informed about lifestyle changes (e.g., nutrition, physical exercise, sleep) that can facilitate brain recovery. To improve learning and consolidation, the education components are accompanied by colorful and comic cartoons [28].
2. Cognitive exercises: According to previous work, using cognitive exercises with game-like features can be useful for supporting treatment engagement and restoration of cognitive functions among people with SUDs [29]. These game-based exercises include such things as Stroop-like tasks [30], spot the differences game [31], and target cancelation tasks [32]. In NEAT, these games are used not only as a way of practicing and improving specific brain functions, but also for enhancing understanding of the brain functions discussed (e.g., as a way of demonstrating what is meant by “inhibition” or “working memory”) and supporting metacognitive awareness (the next component).
3. Metacognitive training: Metacognition awareness [33, 34] is supported through self-monitoring in between sessions and group discussions about the cognitive functions involved in each brain exercise and the role that these functions (and the associated compensatory strategies) play in their day-to-day functioning, craving management, and substance use recovery. This component targets insight towards the cognitive foundations of the training program and aims to enhance motivation to implement skills and generalization of learning.
4. Compensatory strategies: The main purpose of these components is to provide specific cognitive strategies for supporting optimal cognitive functioning and compensating for deficits. For example, providing mnemonic aids and goal management training for memory- and planning-related problems [35, 36]. These components are integrated with the cognitive exercises discussed above to provide a thorough understanding and awareness of the role of specific cognitive functions in their lives.
5. Brain planner: Training participants to keep records of their daily activities, including both retrospectively (e.g., recording past events) and prospectively (e.g., planning future activities and goals), is another critical aspect of NEAT. Planner use can support optimal daily functioning and the use of higher-order cognitive functions such as self-monitoring and planning (which are also supported by compensatory strategies discussed above) but can also help compensate for deficits in these cognitive domains and others (i.e., memory deficits) [37]. The use of a planner also supports compliance with NEAT and other substance use treatment activities. Each training session of NEAT has time dedicated to learning and practicing using the planner.

Based on the nature of the intervention, there are no anticipated serious adverse events (SAEs), adverse events (AEs), or harms from the intervention. In case of any minimum AEs during the assessments or intervention, the study investigators will be involved to mitigate any risks or consequences and the details will be documented and reported to the LIBR leadership team and the IRB committee (WIRB) according to the WIRB protocols. The reason for dropout for participants who discontinue or deviate from the intervention protocols will be collected for further exploratory analyses.

### Measures

**Fig. 4:**
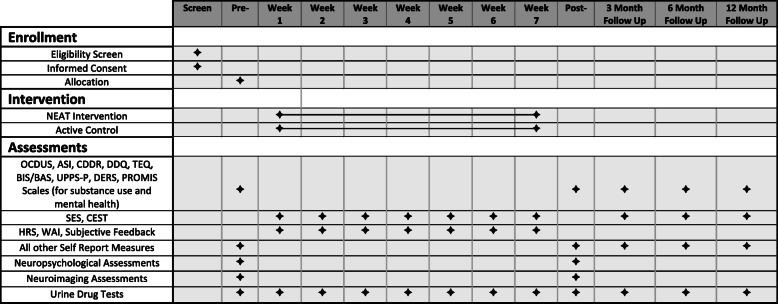
Schedule of enrollment, interventions, and assessments. This figure indicates the assessments or interventions completed for screening, pre-intervention, weekly during the completion of the intervention, post-intervention, and 3- and 6-month follow-up. Participants are randomized in groups of 9–12 to complete either neuroscience-informed psychoeducation (NEAT) or active control intervention. *OCDUS*, Obsessive Compulsive Drug Use Scale; *ASI*, Addiction Severity Index; *CDDR*, Customary Drinking and Drug Use Record; *DDQ*, Desire for Drug Questionnaire; *TEQ*, Traumatic Events Questionnaire; *BIS/BAS*, Behavioral Inhibition System/Behavioral Approach Scale; *UPPS-P*, UPPS Impulsive Behavior Scale; *DERS*, Difficulties in Emotion Regulation Scale; *PROMIS*, Patient-Reported Outcomes Measurement Information System; *SES*, Services Engagement Scale; *CEST*, Client Evaluation of Self and Treatment; *HRS*, Homework Rating Scale; *WAI*, Working Alliance Inventory; *PROMIS-CA*, PROMIS Cog Abilities; *PROMIS-CG*, PROMIS Cog General

Table 1 indicates the list of self-report, behavioral, and neuroimaging assessments included in the protocol (refer to Fig. 4 for the timing of assessments). We will use the total score of the Obsessive Compulsive Drug Use Scale (OCDUS) [21] as a primary outcome measure for drug craving in the past week. It is a 12-item questionnaire consisting of three factors including thoughts about drugs and interference, desire and control, and resistance against thoughts and intention to use drugs that compose a total score. The remaining questionnaires and tasks used as secondary and exploratory outcome measures are described in Fig. 4.

**Table 1:** Diagnostic, demographic, self-report, behavioral, and neuroimaging assessments

Diagnostic and demographic assessment	
Diagnosis	MINI V6.0 [23] or the PRISM-5
History	Assessment of medication history and medication and therapy compliance
Standard self-report scales	
Substance use	Obsessive Compulsive Drug Use Scale (OCDUS) [21]
Substance use	Customary Drinking and Drug Use Record (CDDR) [38]
Substance use	Addiction Severity Index (ASI) [39]
Substance use	Desire for Drug Questionnaire (DDQ) [21]
Substance use	PROMIS Alcohol Use [40]
Substance use	PROMIS Nicotine Dependence [41]
Substance use	PROMIS Substance Use Severity [42]
Mental health	PROMIS Adaptive Scales (depression, anxiety, and anger) [43]
Mental health	Traumatic Events Questionnaire (TEQ) [44]
Mental health	Difficulties in Emotion Regulation Scale (DERS) [45]
Mental health	UPPS Impulsive Behavior Scale [46]
Mental health	Behavioral Inhibition System/Behavioral Approach Scale (BIS/BAS) [47]
Treatment engagement	Services Engagement Scale (SES) [2]
Treatment engagement	Client Evaluation of Self and Treatment (CEST) Engagement [48]
Feasibility/knowledge	Credibility/Expectancy Questionnaire (CEQ) [48]
Feasibility/knowledge	Homework Rating Scale (HRS) [49]
Feasibility/knowledge	Working Alliance Inventory (WAI) [50]
Cognitive functions (subjective)	PROMIS Cog Abilities (PROMIS-CA) [22]
Cognitive functions (subjective)	PROMIS Cog General (PROMIS-CG) [22]
Behavioral assessment	
Neuropsychological	NIH Toolbox Cognitive Assessment Battery [51]
Neuropsychological	Iowa GamblingTask [52]
Neuropsychological	Delayed Discounting [53]
Neuropsychological	Emotional and Classic Go/NoGoTasks [54]
Neuropsychological	Classic version of the go/no-go task [55]
Neuroimaging assessments	
Neuroimaging	T1-W sagittal MP-RAGE
Neuroimaging	fMRI resting state
Neuroimaging	fMRI monetary incentive delay [56]
Neuroimaging	fMRI stop signal [57]
Neuroimaging	fMRI drug cue reactivity [58]
Neuroimaging	T2-W sagittal
Neuroimaging	T2-W axial FLAIR
Neuroimaging	Diffusion tensor imaging
Laboratory assessments	
Substance use	Urine drug test
Biomarker assessment	Blood sample test

Biological specimens (blood and oral and mouth microbiome) data will be collected in the baseline for further exploratory analysis on the predictive role of biomarkers on response to interventions. Less than 150 mL of blood will be collected per subject, which is well within the safety limit of ~ 450 mL per blood draw. Blood will not be drawn from subjects with a hematocrit below 30%. A trained phlebotomist will obtain blood samples in the morning of one of the visits, or at a time convenient for the participant. Participants will also be asked to provide microbial samples during the biomarker session.

For blood samples, plasma, serum, PBMCs, and genetics will be transported to and processed at the University of Oklahoma Integrative Immunology Center (IIC) Laboratories. Plasma, serum, and genetic samples will be stored in secure freezers at − 80 °C. Freezers will be maintained in a specially equipped room with emergency backup power and an automated telephone alarm system that is programmed to call in case of failure. Additional aliquots of samples will be stored at − 80 °C should repeat analyses be required at a later date. PBMCs will be stored in liquid nitrogen dewars with liquid level monitors and alarms in a secure room at the University of Oklahoma IIC Laboratories.

Participants will also be asked to provide microbial samples during the biomarker session. All participants will be asked to provide forehead, mouth, and stool samples. A research assistant will provide the participant with an all-in-one sample collection kit system for collecting, stabilizing, transporting, and purifying samples which include cotton swabs, tubes labeled by body area, and a one-page sheet with step-by-step instructions. Once a participant has obtained all samples and provided them to the research assistant, the research assistant will label each tube with the subject’s ID number and date of sample. Once the sample has been properly labeled, it will be placed in a freezer in a locked office. The microbiome samples will be transported to the University of Oklahoma IIC Laboratory for DNA extraction and long-term storage in secure freezers at − 80 °C. Analysis of the de-identified samples will be conducted at the University of California-San Diego.

### Data management

Confidentiality will be maintained by keeping identifying information (such as names and phone numbers) in secure servers. Except for a contact form, research data will be identified by a subject number rather than by name. An alpha numeric system will be used for the identification of subjects. All hard copies of study information will be kept in locked file cabinets inside a locked office. All electronic study information will be stored on secure servers with appropriate access controls. Only study personnel will have access to the identifying data. The WIR staff will be aware of who is participating in the research study, as they will know who is attending this group versus the other WIR activities planned at that time. In addition, these treatment providers may insert into their treatment records that they attended these treatment sessions. However, treatment program staff will not be privy to any of the information provided to the research study personnel via interview, behavioral, or self-report assessments. Subject identity will not be disclosed to any person who is not authorized to see this information, except in the event of a medical emergency or if required by law. Paper copies of consents, screening forms, the research privacy form and any other forms, and testing results or papers containing personally identifiable information (PII) will be stored in locked cabinets at the Laureate Institute for Brain Research (LIBR). Any electronic data will have all identifiable information encrypted and be stored on a password-protected database on a secure server managed by LIBR.

Participants will be told that their information provided during the course of the study will remain confidential within the research study team. However, given the nature of the treatment groups held at WIR, confidentiality concerning their participation in the study cannot be guaranteed. Family members will be allowed to be present and discuss the consenting process with the participant if requested. The consent mentions the biological samples collected in this study will be stored for future research. Participants are also informed that the data collected as part of this study, including interviews and medical information, research test results, and biological specimens, may be shared with collaborators (researchers at other nonprofit research institutions, government or commercial organizations), and any data shared will not include the name or other personally identifiable information. The consent form is available from the corresponding author on request. Additionally, Certificates of Confidentiality (CoCs) will be asked from the National Institute of Health (NIH) to protect the privacy of research subjects by prohibiting disclosure of identifiable, sensitive research information to anyone not connected to the research except when the subject consents or in a few other specific situations.

### Statistical analysis

Categorical variables will be summarized by frequency and proportion, and continuous variables by mean and standard deviation (SD), or by median and inter-quartile range if the distribution, appeared skewed even after simple (e.g., log) transformations, for all participants and for participants in each treatment arm. Data normality will be evaluated by quantile-quantile (QQ) plot and Shapiro-Wilk test.

Treatment effects will be compared by the intention-to-treat (ITT) principle, i.e., all subjects who were randomized will be included in the final analysis, regardless of their adherence to any of the interventions. In order to detect changes in the variables recorded for the primary and secondary outcomes, mixed-effects model analyses will be performed using fixed-effects of time (pre-, post-intervention time points) as a within-subject factor and condition (intervention vs. control) as a between-subject factor; within-subject dependency will be captured by either random cluster and/or subject intercepts or within-subject correlation structure (e.g., 1st-order autoregressive or exponential power), whichever Akaike or Bayesian information criterion (AIC or BIC) favor. Mixed-effects models allow using all available observations and, without the need of imputation, provide unbiased estimates under the missing at random mechanism, a plausible assumption for randomized trials, and are known to be more powerful than any ad hoc imputation [59]. We include age and IQ as covariates. The inclusion of other covariates will be determined by AIC and BIC. Subgroup analyses will be conducted according to the baseline neuropsychological and fMRI characteristics. Significance level will be set at 0.05 level for the primary outcome and hypothesis; Bonferroni correction will be applied to all pre-post comparisons for all secondary outcomes, and Benjamini-Hochberg’s procedure to control false discovery rate at 5% level for all the exploratory analyses. For each outcome measure (primary, secondary, and exploratory), effect sizes of Cohen’s *d* and *f* will be computed. The effect sizes, along with the attrition rate at each time point, can be used to guide future study design.

## Discussion

In many people with substance use disorders, the lack of self-awareness on how their brains behave to develop cravings and initiate relapse is a complication that hampers efforts for abstinence and delays progress in recovery [52, 60]. Given this deficit, this group of patients are well-equipped to manage their cravings, and may deny drug-related problems and underestimate the need for treatment [61, 62]. Enhancing self-awareness by training neurocognitive skills to help patients to deal with drug craving and other cognitive consequences of chronic drug use is the aim of this study that was described in detail above. In this work, we developed a new neurocognitive rehabilitation program (NEAT) that addresses neurocognitive deficits reported by previous studies in substance users [11, 63, 64]. The unique aspect of NEAT is using interactive and engaging structure (including cartoons, brain awareness games, and real-life scenarios) to train the complex content of neurocognitive functions (e.g., interoception, episodic future thinking, inhibition) and metacognitive and compensatory skills (e.g., self-monitoring, goal management training).

We assume that compared to the control intervention, the patients who receive the NEAT program indicate positive changes in their roadmap for recovery during the course of the study. These changes are measured by using clinical, psychological, and neuropsychological assessments as well as neuroimaging techniques.

Fewer studies before have indicated the effectiveness of metacognitive awareness and cognitive rehabilitation interventions in alcohol and substance users, in terms of craving control, improved cognitive functions, and better treatment outcomes (e.g., treatment adherence, length of abstinence) [65–71]. Although in all these studies, the promising results are viewed as a sign of improved regulatory control of top-down system over bottom-up system, but no evidence exists for underlying neural substrates. Therefore, our study is the first attempt that applies a broad range of assessments including neural components to monitor the effect of a cognitive rehabilitation program in substance users. We also consider the effect of the intervention on real-world functioning (e.g., craving management and social and emotional competency) to provide more data on the ecological validity. Randomized allocation of patients in two groups, employment of an active control condition, blinding outcome assessors, using a fully manualized intervention, and monitoring the long-term effect of intervention during 1-year follow-up are the most important strengths of the present study.

In this study, we are expecting to face the following limitations: (1) participant withdrawal due to schedules changing or phasing up in the program, (2) scheduling of other appointments (i.e., visiting a physician) during Brain Gym times, (3) participants not completing suggested homework or not fill the brain planner, (4) participants absconding from the WiR program or being terminated from the program, and (5) follow-up problem and lack of data about those participants once they are discharged from WiR.

To conclude, the effects of a multi-component neurocognitive rehabilitation and awareness program termed “Neurocognitive Empowerment for Addiction Treatment” (NEAT) on psychological, neurocognitive, and structural and functional neuroimaging measures will be examined in a group of female opioids and or methamphetamine users in the context of addiction recovery. If the results of this study prove the efficacy of NEAT as it is expected, then a larger trial will be needed to provide confirmatory evidence to support the next step for transferring the intervention to the clinical practice and embedding NEAT into the routine therapeutic programs as a supplementary intervention.

### Trial status

The present trial is ongoing. Recruitment commenced in January 2019. Data collection is predicted to be completed by the end of 2021.

## Supplementary Information

**Additional file 1.** CONSORT 2010 checklist of information to include when reporting a randomised trial*.

**Additional file 2.** SPIRIT 2013 Checklist: Recommended items to address in a clinical trial protocol and related documents*.

## Data Availability

The data that will be collected in this study will be available from the principal investigator on reasonable request.

## Abbreviations

ASI: Addiction Severity Index
BIS/BAS: Behavioral Inhibition System/Behavioral Approach Scale
CEST: Client Evaluation of Self and Treatment
CEQ: Credibility/Expectancy Questionnaire
CDDR: Customary Drinking and Drug Use Record
DCM: Dynamic model of craving
DDQ: Desire for Drug Questionnaire
DERS: Difficulties in Emotion Regulation Scale
HRS: Homework Rating Scale
MINI: Mini-International Neuropsychiatric Interview
MUD: Methamphetamine use disorder
NCDs: Neurocognitive deficits
NEAT: Neurocognitive Empowerment for Addiction Treatment
OCDUS: Obsessive Compulsive Drug Use Scale
OUD: Opioid use disorder
RCT: Randomized controlled trial
SES: Services Engagement Scale
TAU: Treatment-as-usual
TEQ: Traumatic Events Questionnaire
WAI: Working Alliance Inventory
WIR: Women in Recovery
